# X-ray refinement significantly underestimates the level of microscopic heterogeneity in biomolecular crystals

**DOI:** 10.1038/ncomms4220

**Published:** 2014-02-07

**Authors:** Antonija Kuzmanic, Navraj S. Pannu, Bojan Zagrovic

**Affiliations:** 1 Department of Structural and Computational Biology, Max F. Perutz Laboratories, University of Vienna, Campus Vienna Biocenter 5, A-1030 Vienna, Austria; 2 Biophysical Structural Chemistry, Leiden University, PO Box 9502, 2300 RA Leiden, The Netherlands

## Abstract

Biomolecular X-ray structures typically provide a static, time- and ensemble-averaged view of molecular ensembles in crystals. In the absence of rigid-body motions and lattice defects, B-factors are thought to accurately reflect the structural heterogeneity of such ensembles. In order to study the effects of averaging on B-factors, we employ molecular dynamics simulations to controllably manipulate microscopic heterogeneity of a crystal containing 216 copies of villin headpiece. Using average structure factors derived from simulation, we analyse how well this heterogeneity is captured by high-resolution molecular-replacement-based model refinement. We find that both isotropic and anisotropic refined B-factors often significantly deviate from their actual values known from simulation: even at high 1.0 Å resolution and *R*_free_ of 5.9%, B-factors of some well-resolved atoms underestimate their actual values even sixfold. Our results suggest that conformational averaging and inadequate treatment of correlated motion considerably influence estimation of microscopic heterogeneity via B-factors, and invite caution in their interpretation.

## Abstract (web summary)

The structural heterogeneity of a biomolecular crystal structure is typically captured using atomic B-factors, determined during structure refinement. Here, the authors use molecular dynamics to show that this strategy is flawed, and that crystallographic B-factors underestimate structural heterogeneity.

## Graphical abstract

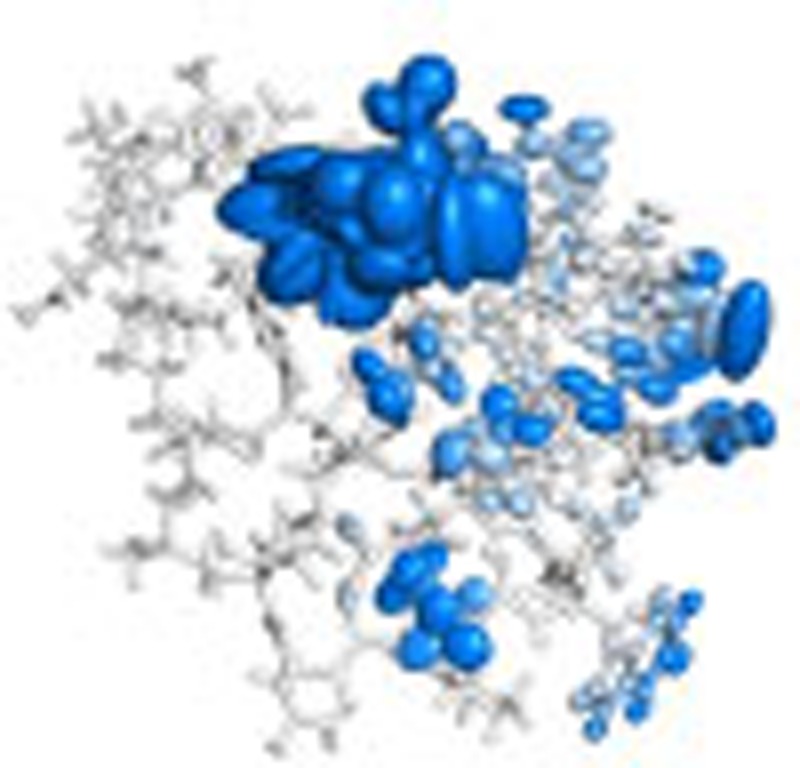

X-ray crystallography is the most widely used experimental method for biomolecular structure determination with over 85% of structures currently deposited in the RCSB Protein Data Bank (PDB) having been solved this way^1^. However, while coordinate accuracy of biomolecular X-ray structures routinely reaches sub-angstrom levels, crystallographic experiments cannot directly probe the dynamics of biomolecules and can capture their structural heterogeneity only indirectly^2^,^3^. As crystallographic observables are averages over both time and space, X-ray structures typically give just a static, average view of the dynamic, structurally heterogeneous ensembles contained in crystals^3^,^4^. On the other hand, the necessity to study dynamics and structural heterogeneity and incorporate them into our understanding of biomolecular function has long been recognized^5^,^6^,^7^. Moreover, dynamics directly influences the very process of structure determination as structural averages derived from diverse ensembles may exhibit certain features, which are not necessarily representative of the true microscopic reality and are a direct artifact of averaging^4^,^8^,^9^,^10^. While these effects have been well studied in the area of biomolecular nuclear magnetic resonance (NMR) and other spectroscopic methods, they are still largely underexplored when it comes to scattering experiments with some exceptions^4^,^9^,^10^,^11^,^12^,^13^.

The pre-eminent way of obtaining and analysing microscopic dynamics and heterogeneity in biomolecular X-ray crystallography is through isotropic and anisotropic B-factors (temperature or Debye–Waller factors)^14^,^15^. Ideally, B-factors capture the attenuation of X-ray scattering due to thermal motion and can be quantitatively related to variances of atomic positional distributions. They have been used in a wide variety of applications ranging from the assessment of the dynamics of proteins^16^ to the prediction of their flexibility^17^,^18^ and thermal stability^19^ to the calculation of quasi-harmonic entropy^20^ and the comparison to other estimates and measures of protein dynamics (for example, fluctuations from elastic network models,^21^,^22^ NMR^23^ and molecular dynamics (MD) simulations^11^,^24^,^25^). Recently, we have shown how one can use B-factors to derive an upper limit on the average pairwise atom-positional root-mean-square deviation (RMSD) between the conformers in a biomolecular crystal^26^. Moreover, we have also shown that B-factors can be exploited to correct errors in distances between average atomic positions caused by thermal fluctuations^27^. Importantly, it is well known that B-factors do not report solely on the fluctuations of atoms but also on other factors such as crystal lattice defects, rigid-body motions, occupancy levels or refinement artifacts^28^,^29^,^30^. These limitations notwithstanding, B-factors are commonly used to distinguish mobile parts from rigid ones in X-ray structures and are in the ideal case taken as a quantitatively accurate measure of positional fluctuations of atoms.

In a classical study, Kuriyan *et al.*^11^ observed that isotropic B-factors obtained by the least-squares refinement of average structure factors calculated from MD simulations underestimate atom-positional deviations present in the simulations. However, likely due to the computational limitations of the time, the authors examined a single protein and assumed non-correlated behaviour between and within unit cells in the crystal, ignored the role of crystal contacts and had to resort to roto-translational fitting of conformers, all of which could have contributed to the deviation between actual and refined B-factors. The same can be claimed about the difference between the least-squares refinement only available at the time and the maximum likelihood refinement used in most modern refinement programs^31^,^32^,^33^. Second, Garcia *et al.*^12^ have used several analytically tractable models and a 400-ps MD simulation to show that multimodal, anharmonic motion can lead to experimental B-factors that are too low compared with the actual mean-square displacements of atoms. Finally, Janowski *et al.*^34^ have recently performed a sizable set of unrestrained large-scale MD simulations of 36 unit cells of a helical decapeptide crystal with low solvent content for which they have observed excellent agreement with the experimental data at the level of B-factors, except in cases where the residues were present in two distinct conformations. Owing to the high degree of crystal packing, however, the overall level of dynamics exhibited by the decapeptide in this study was relatively low and was in this sense reminiscent of a small molecule crystal.

Here we investigate how averaging over many structurally different conformers in a protein crystal affects isotropic and anisotropic B-factors obtained in crystallographic refinement using state-of-the art simulation and refinement approaches. In particular, we analyse a system in which, unlike in a typical crystallographic experiment, one has full control over microscopic variability and can directly manipulate it. More specifically, we use MD simulations to create a microscopic model of a crystal containing villin headpiece domain with predesigned structural heterogeneity. Villin headpiece is a 35-residue 3-helix bundle protein and is one of the most widely studied model systems in protein biophysics due to its small size and fast-folding properties^35^. We generate extensive simulations of a crystal containing 216 explicitly modelled molecules in which all contributions to B-factors other than thermal atomic motions have been eliminated. Specifically, we achieve high structural diversity through MD simulations at 350 K, but keep 60% of atoms in each molecule fixed in place by position restraints, thus limiting their dynamics and eliminating rigid-body motions of the protein at the same time. The remaining 40% of atoms in each molecule are free to explore various conformations. We subsequently calculate structure factors for the simulated crystal, average them and then solve the structure by molecular replacement (MR) and several different refinement procedures. Finally, we analyse the resultant model and compare the refined harmonic isotropic or anisotropic B-factors against their MD-based counterparts, which in turn represent atomic fluctuations that are actually present in the system used for the generation of structure factors. Such internally self-consistent comparison between the refined model and the actual microscopic ensemble known from the simulation allows us to study how accurately X-ray refinement captures the true structural diversity of a biomolecule present at the microscopic level.

## Results

### Heterogeneity of the simulated villin headpiece crystal

**Figure 1:**
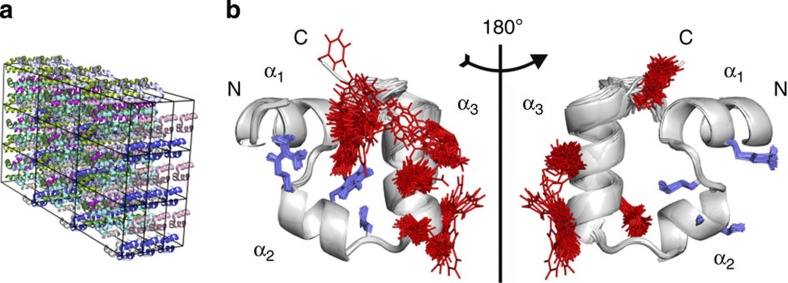
Villin headpiece domain crystal and its heterogeneity. (**a**) Simulated 3 × 3 × 3 crystal of the villin headpiece domain (2F4K). All the structures at symmetrically equivalent positions in the unit cells are coloured the same. (**b**) 100 structures taken at random from the complete MD simulation illustrate the difference in mobility between the parts of the molecule, which were position-restrained (α_1_- and α_2_-helices with selected side chains shown in blue: Arg14, Phe17, Leu20 on the left; Lys7, Met12, Ser15, Ala16 on the right) and the unrestrained ones (α_3_-helix with selected side chains shown in red: Leu22, Trp23, Gln26, Lys30, Phe35 on the left; Trp23, Gln25, His27, Leu34 on the right).

With a total of 27 unit cells, 216 protein copies and a total of 118,752 atoms including solvent, the villin headpiece crystal studied here is to the best of our knowledge one of the largest biomolecular crystals simulated to date using atomistic MD (Fig. 1a). During MD simulation, residues 1–21 (encompassing helices α_1_ and α_2_) remain largely immobile as a consequence of strong position restraints, while the rest of the protein (including helix α_3_) explores various conformations freely. In addition to providing an internal control, position-restraining 60% of atoms in the molecule has enabled us to minimize the effects of rigid-body motions and lattice irregularities on B-factors, which are known to mask the true dynamics in proteins^28^. Finally, as our main objective was not to analyse the true level of microscopic heterogeneity in the villin headpiece crystal, but rather generate a structurally diverse ensemble, the simulations were run at 350 K. The inclusion of position restraints and explicit solvent in the crystal, on the other hand, ensured that the final structural ensemble, although not necessarily matching the real microscopic ensemble of villin headpiece in the crystal, is still physically realistic to a high degree.

**Figure 2:**
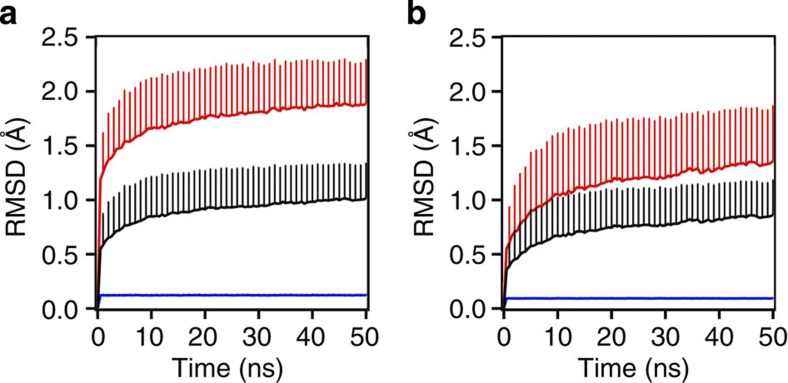
Structural changes of individual monomers in the crystal during simulation. (**a**) Average all-atom RMSDs over time, and (**b**) backbone RMSD calculated for residues 1–35 (black line), residues 1–21 (restrained, blue line) and residues 22–35 (unrestrained, red line) over time. The values shown have been averaged over 216 monomers in the crystal every 500 ps and displayed together with their s.d. Only crystal-specific alignment has been applied before the calculations.

Despite the rather dense packing of the crystal (Matthews coefficient of 1.82 Å^3^ Da^−1^ and solvent content of 32.27%), the protein achieves a high level of structural diversity as illustrated in Fig. 1b. As can be seen from 100 structures chosen randomly from the complete MD simulation, the two parts of the protein that were treated differently in the simulations adopt a contrastingly varied number of different conformations. This is illustrated in the figure by explicitly drawing only the large side chains of amino acids such as Arg, Phe, Trp, His and Glu, with the C-terminal Phe displaying the highest level of conformational heterogeneity. The varying degree of structural diversity in the simulated ensemble is also seen from atom-positional RMSD, which was calculated against the starting structure for each monomer over time and averaged over 216 monomers in the crystal (Fig. 2a). The average RMSD increases rapidly to ~0.5 Å in the first 500 ps of the simulation for all-atom calculations (~0.35 Å for the backbone, Fig. 2b) and then rises slowly to ~1 Å over the next 50 ns (~0.75 Å for the backbone, Fig. 2b) with the maximal values reaching up to 2.4 and 2 Å for all-atom and backbone calculations, respectively. These values are somewhat higher than the ones typically reported for MD simulations of protein crystals^36^,^37^ and they actually approach the average RMSD values of native villin structures in solution simulations^35^,^38^. On the other hand, while the restrained segment maintains a very low average RMSD (~0.1–0.2 Å), the RMSD curves for the unrestrained segment exhibit features similar to the curves calculated for the complete molecule, reaching a maximum of ~4 and 3 Å for all-atom (Fig. 2a) and backbone (Fig. 2b) calculations, respectively.

Calculation of atom-positional root-mean-square fluctuations (RMSFs, Supplementary Fig. 1) further highlights the contrast between the restrained and unrestrained parts of the molecule. As expected, the restrained segment exhibits very little displacement of its coordinates with both all-atom and the backbone RMSFs maintaining a very low value of ~0.1 Å. However, the unrestrained segment shows a much higher level of mobility with RMSFs reaching maximum values of ~5.6 Å for all-atom calculations and ~3.6 Å for backbone ones. These extreme values are observed for the C-terminal phenylalanine due to its less constrained position in the molecule. Importantly, all the values reported here are expectedly high, given our simulation setup, and they further indicate that we have successfully created a diverse ensemble of structures that is not affected by rigid-body motions or lattice defects. The latter is attested by the fact that all of the above calculations, including those for the rigid part of the molecule, were performed without least-squares fitting (see Methods for details).

### Comparison of actual and refined B-factors

Crystallographic structure factors were calculated for each individual frame of the simulated crystal and then averaged and used further in MR using as a starting model either the X-ray structure of villin headpiece or the average simulated structure (see Methods for details). The two solutions generated for these models by MR were used in the refinement, which differed additionally when it comes to the application of geometric restraints and the type of B-factor refinement used. Different combinations of these choices resulted in eight refinement procedures all of which give reasonably low *R* and *R*_free_ factors (~8–11%) for the resolution range of 37.5–1.0 Å and low RMS values for the stereochemical quantities, if applicable (Table 1). Overall, anisotropic refinement always performs better than isotropic refinement as indicated by *R* and *R*_free_ factors, which are typically lower by ~0.02 in the former case. On the other hand, the presence or the absence of geometric restraints makes little difference regardless of the starting MR model used. However, despite a low *R*_free_ factor, the lack of geometric restraints greatly distorts the geometry of certain residues (for example, His27, Glu31, Leu34 and Phe35) even when the experimental structure is used as a model in MR. Finally, in order to improve our models further, we have performed 15 additional cycles of refinement with hydrogen atoms in riding positions. This was only possible for restrained MR refinement, which used the experimental structure as a model. Namely, *refmac* was unable to add hydrogens to final models of unrestrained refinements due to nonphysical residues and scattered atoms. Moreover, during the restrained refinement of the average structure, *refmac* restores the nonphysical geometry to its ideal state, which defies the purpose of using such a model to begin with. A total of 25 cycles of geometrically restrained, anisotropic refinement without hydrogens and using the experimental structure as a model, followed by additional 15 cycles with hydrogens in riding positions have ultimately resulted in a significant reduction in *R* and *R*_free_ factors with the final values of 5.57 and 5.85%, respectively (Table 1). Very similar results were obtained from averaged structure factors that were calculated by using a finer grid spacing of 0.225 Å in electron density sampling (Supplementary Table 1), or from structure factors calculated for the average density map obtained by *mapman*^39^ (Supplementary Table 2).

**Figure 3:**
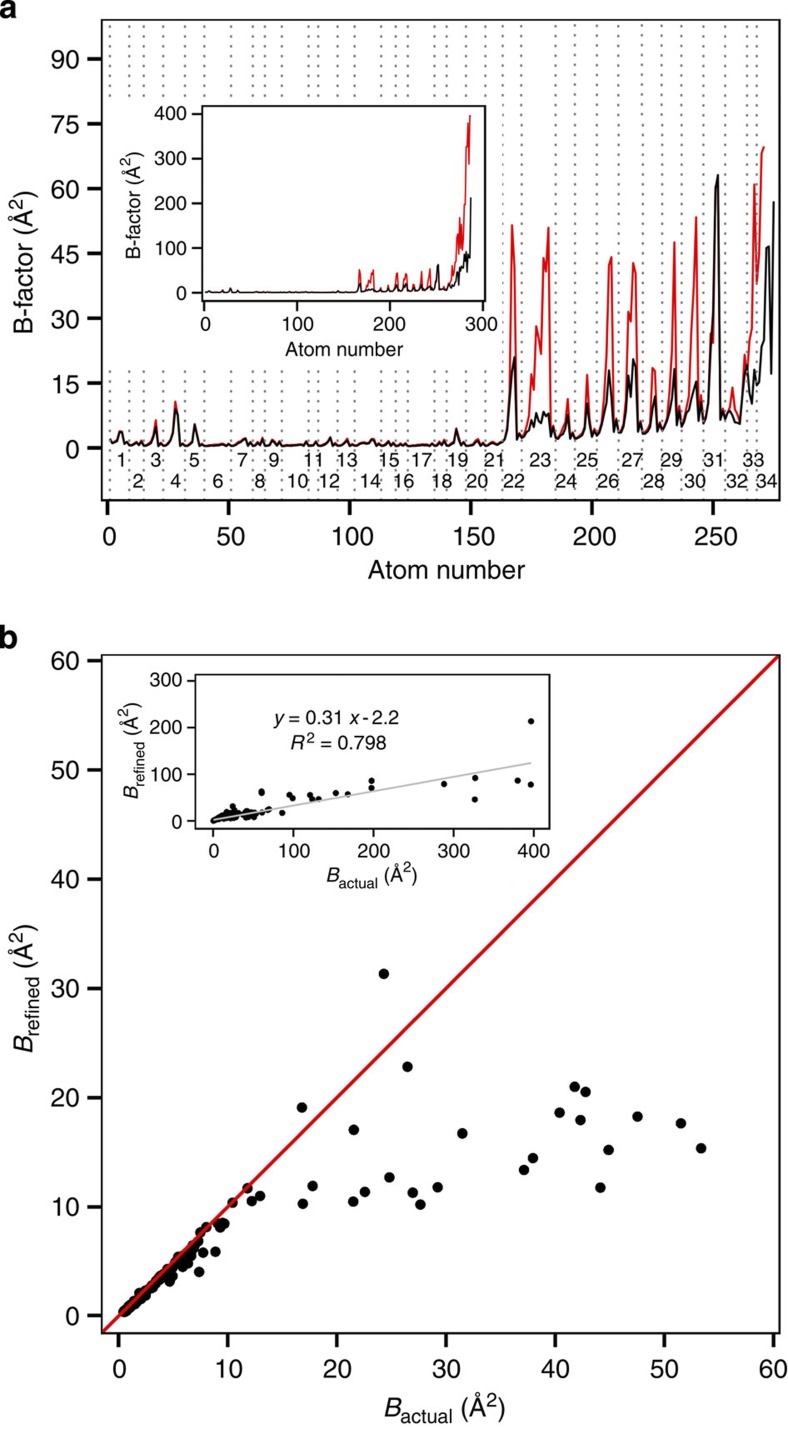
Comparison of actual and refined B-factors. (**a**) Comparison of heavy-atom B-factor profiles calculated directly from MD simulation (red line) with those obtained by refinement (black line) for residues 1–34 (main figure) and 1–35 (inset). Every residue in the main figure has been labelled and encased with grey dotted lines. (**b**) The refined heavy-atom B-factors versus the actual simulated ones. The data shown in the plot have been limited to the actual B-factors smaller than 60 Å^2^ with the identity line shown in red. The full data set is shown in the inset along with the trend line (grey), its analytic expression and *R*^2^.

Importantly, none of the performed refinement procedures manage to capture the true heterogeneity of the underlying structural ensembles, known from simulation, either through isotropic or anisotropic B-factors (Fig. 3). Even for the model with the lowest obtained *R*_free_ factor (5.85%) and an ideal geometry, the refined B-factors capture the actual atomic displacements with only sporadic success (Fig. 3a). Namely, while they accurately describe the diversity of the restrained part of the molecule, the discrepancy exhibited for the side chains of the mobile segment is striking. For example, large differences of 20–40 Å^2^ between the actual and the refined B-factors can be observed for residues Trp23, Gln26 and His27, all of which are well resolved and only moderately dynamic. In particular, for some atoms the difference between the two values is more than sixfold. For example, the refined B-factor of C_η_ in Trp23 is 8 Å^2^, while the actual value for the same atom is 51 Å^2^ (note that the term ‘actual’ as used throughout the text refers to the actual microscopic ensemble present in the simulation and not to the real ensemble in the physical crystal of villin headpiece). The most prominent difference of ~340 Å^2^ is observed for the C-terminal Phe; however, due to its high mobility (Fig. 1), this disagreement is not surprising. On the other hand, Glu31 is the only amino acid in this segment with high atomic displacements that were overestimated, albeit only marginally. Overall, these discrepancies indicate that a large degree of information on the flexibility and motions present in the molecule is lost in the refinement process. Finally, the RMS-average deviation between the actual B-factors and the refined ones as calculated over all atoms (Table 1) exceeds 35 Å^2^ for all types of refinement with somewhat lower values seen for anisotropic refinement. In addition, if the maximal difference between the two B-factor profiles is compared for different refinement setups, no major variability is seen with the value of ~300 Å^2^ in all cases.

How does the magnitude of the refined B-factors depend on their actual values (Fig. 3b)? For the actual B-factors below ~10 Å^2^, there is very little deviation between them and the refined values. On the other hand, in the range of 10–60 Å^2^, where one would still expect a reasonable agreement between the compared values, the refinement leads to an underestimation of the actual B-factors by 85% and an approximate linear relationship between the actual and the refined values. Furthermore, if the correlation between the two is calculated for the complete data set (Fig. 3b, inset), the refined B-factors underestimate the actual ones by ~70% on average, with the Pearson *R*^2^ correlation coefficient of 0.798 between them. However, in this range, the fit is heavily influenced by a few points exhibiting extreme values for the simulated B-factors and its predictive power in the range of low B-factors is relatively poor. We have also compared refined anisotropic B-factors with the simulated ones—that is, the comparison was performed at the level of individual anisotropic displacement parameters (ADPs) (Supplementary Fig. 2). The results agree with what was observed for isotropic B-factors. Essentially, the refined profiles for the restrained part of the molecule show a good agreement with the simulated ones, while the unrestrained part again displays large discrepancies between values in all ADPs. While the differences are most prominent for the C-terminal Phe, they occur on a smaller, but still prominent, scale for Leu22, Trp23, Gln26, His27, Nle29 and Lys30. However, in the case of Glu31, the only residue for which isotropic B-factors were overestimated, the agreement is poor at the level of all individual atomic ADPs (Supplementary Fig. 2).

### Effects of heterogeneity on the refined density map

**Figure 4:**
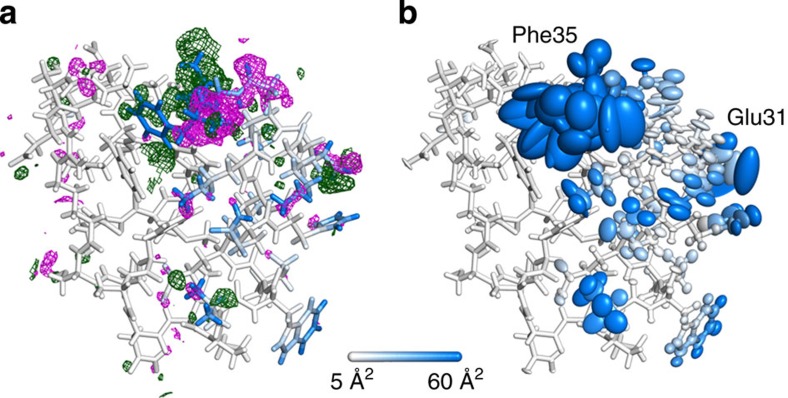
Final refined model and its electron density difference map. Refined electron density difference map (*F*_o_−*F*_c_) and its accompanying structural model (**a**) for the restrained anisotropic refinement solved by molecular replacement using the simulation starting structure as the initial model. The same final model with its anisotropic ellipsoids is also displayed (**b**). The electron density maps have been calculated by CCP4 programs *fft*^67^ and *mapmask*, contoured at 3σ and displayed with a cutoff of 2 Å from each atom. The negative density is shown in magenta and the positive one in green. Each atom has been coloured by the simulated B-factor associated with it (the scale changes continuously from white to blue with cutoffs at 5 and 60 Å^2^).

Is the discrepancy between the refined and the actual B-factors reflected in the refined electron density map? We have calculated the difference map (*F*_o_−*F*_c_) for the chosen refinement setup described above and displayed it with its final structural model (Fig. 4a). In addition, we have colour-coded the actual B-factors with a scale changing continuously from white (<5 Å^2^) to blue (>60 Å^2^). One quickly notices the presence of large portions of positive and negative electron densities surrounding the highly mobile C-terminal phenylalanine indicating that this segment has been modelled poorly. On the other hand, several extremely mobile and structurally heterogeneous parts of the molecule appear to be surprisingly well described by the model as can be seen from the low level of unmodelled or wrongly modelled electron densities. Moreover, despite the significant structural heterogeneity of the ensemble, the refined anisotropic B-factors adopt extremely large values only for Glu31 and Phe35 (Fig. 4b). Nevertheless, real-space correlation coefficients^40^ remain very close to 1.0 for the first 33 residues and then drop to ~0.8 for the remaining two (Supplementary Fig. 3).

**Figure 5:**
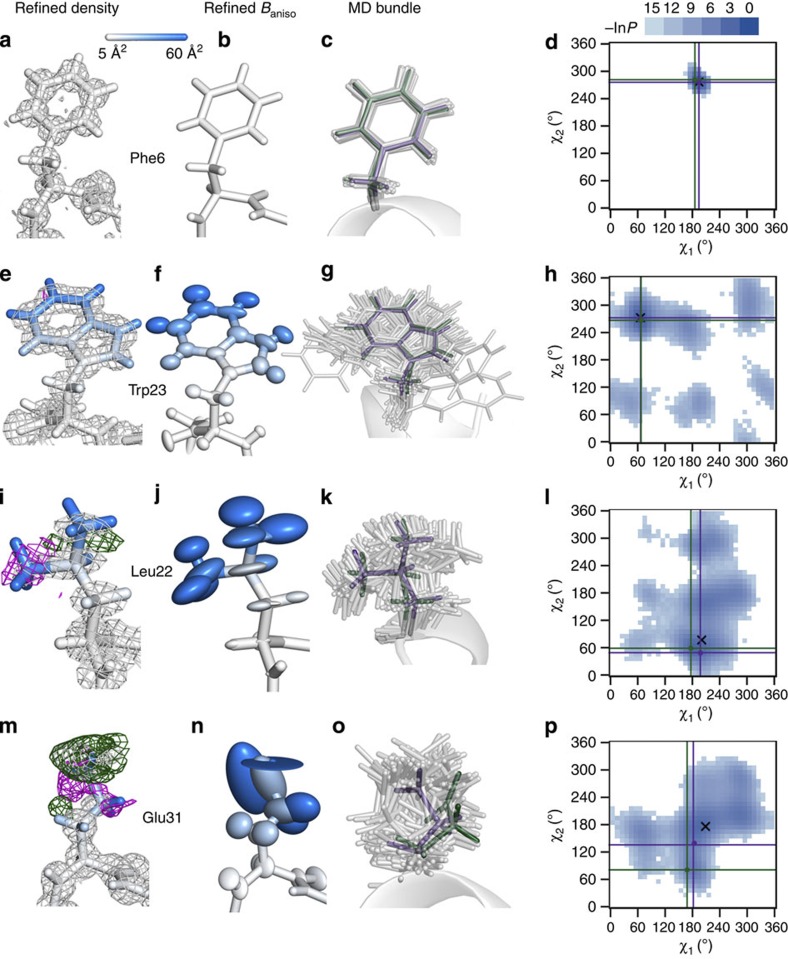
Electron density maps of selected residues and their heterogeneity. Selected residues and their refined electron density maps (2*F*_o_−*F*_c_) are shown together with their difference maps (*F*_o_−*F*_c_) (**a**,**e**,**i**,**m**) for the geometrically restrained refinement that used the starting structure from simulation as a model for molecular replacement and anisotropic refinement of B-factors (shown with the ellipsoids—**b**,**f**,**j**,**n**): Phe6 (**a**–**d**), Trp23 (**e**–**h**), Leu22 (**i**–**l**), and Glu31 (**m**–**p**). 2*F*_o_−*F*_c_ electron density maps have been contoured at 1σ and the *F*_o_−*F*_c_ difference map at 3σ with negative densities shown in magenta and positive ones in green. Both maps are displayed with a cutoff of 2 Å from each atom. All atoms are coloured by the simulated B-factor associated with them (the scale changes continuously from white to blue with cutoffs at 5 and 60 Å^2^). In addition, a bundle of residues from 100 randomly chosen simulated structures is shown (grey), together with the residues from the final refined model (purple) and the starting model (green) (**c**,**g**,**k**,**o**). Only the backbone of the starting model is shown for clarity. Furthermore, statistical free energy profile of χ_1_ and χ_2_ dihedral angles (*F*=*−RT*ln*P*, where *P* is probability) calculated from the simulation is displayed for each residue as a histogram (bin size=10°, legend given in units of RT) with the angles from the final refined and the starting experimental model labelled as purple and green points, respectively, and emphasized with vertical and horizontal lines of the same colours (**d**,**h**,**l**,**p**). The angles from the average structure calculated from the simulation are shown as a black X.

We have further closely analysed electron densities of mobile side chains and compared them with the two dimensional (2D) χ-angle distributions calculated from the simulations (Fig. 5). In addition, a bundle of 100 randomly simulated chosen structures has been added for each residue to illustrate the true heterogeneity of the ensemble. The results for Phe6, which participates in forming the hydrophobic core of the protein, are included to provide a reference point for the more mobile residues (Fig. 5a–d). Overall, Phe6 is refined well with five hydrogen atoms in close proximity of its benzyl side chain (Fig. 5a,b), a very well-ordered bundle (Fig. 5c), and a χ-angle distribution showing a single peak with a very small area around it (Fig. 5d). Importantly, this can be said for all the residues that were position-restrained in the simulation. On the other hand, the refined B-factors of Trp23 agree poorly with the actual ones (side-chain B-factor RMS deviations of 22 Å^2^), while at the same time its refined density does not at all reflect this fact with only a slight negative density for one atom (Fig. 5e,f). Furthermore, in the distribution of Trp23 χ_1_ and χ_2_ angles, one observes a high-density peak centred at ~70° and 270°, respectively, where both the starting and the final models can be found, while other areas in the distribution are populated less frequently (Fig. 5h). Overall, this side chain exhibits a dominant average orientation with rather large displacements around it (Fig. 5g,h). The combination of these features could be enough to create a difference at the level of ensemble RMSF calculations—that is, B-factors—but not necessarily at the density level. A similar situation is seen for residues Gln26 and His27, which exhibit well-resolved densities and at the same time major discrepancies between actual and refined B-factors (see Fig. 3a and also below).

On the other hand, several residues exhibit significant discrepancy between the actual and the refined B-factors; however, their densities are detectably less well refined. For example, Leu22 (Fig. 5i–l) exhibits a poorly refined density around its side-chain methyl groups (Fig. 5i) together with large ellipsoids that cannot capture the full extent of the diversity exhibited by the underlying ensemble (side-chain B-factor RMS deviations of 18 Å^2^) (Fig. 5j,k). If one looks at the χ_1_ and χ_2_ angle distribution, one sees that the final and the starting models are close to the highest peak in the distribution but not as close to the values for the average structure as Phe6 or Trp23 (Fig. 5l). Finally, the only side chain in the mobile region of the molecule whose refined B-factors are overestimated compared with the actual values is Glu31. In this case, the electron density becomes less orderly as one moves away from the backbone (Fig. 5m). This is accompanied by large anisotropic B-factors that apparently do not capture this flexibility well (Fig. 5n; Supplementary Fig. 2). In addition, the distribution of χ angles shows a large spread of values with a poorly populated central peak (also with the average values in its vicinity) (Fig. 5o,p).

### Ensemble heterogeneity is not reflected in electron density

**Figure 6:**
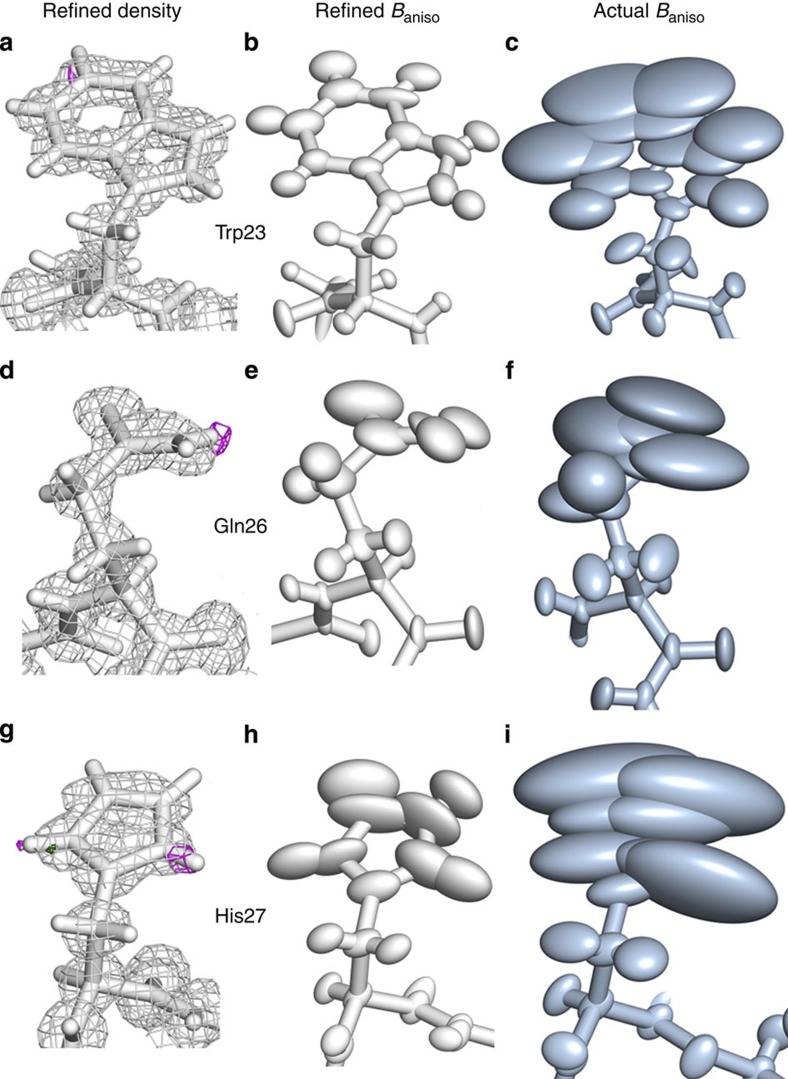
Comparison of the refined anisotropic thermal fluctuations with the actual simulated ones. The final model obtained by molecular-replacement-based, restrained, anisotropic refinement is shown in white with its electron density (**a**,**d**,**g**) and anisotropic B-factor ellipsoids (**b**,**e**,**h**), while the actual simulated average structure with its anisotropic B-factor ellipsoids is shown in slate (**c**,**f**,**i**). All the residues are shown from the same perspective: Trp23 (**a**–**c**), Gln26 (**d**–**f**), and His27 (**g**–**i**).

Finally, we have also compared anisotropic B-factors obtained from refinement with the actual ones calculated from simulation for the residues that exhibit well-refined densities but low matching in B-factor profile—Trp23 (Fig. 6a–c), Gln26 (Fig. 6d–f) and His27 (Fig. 6g–i). A side-by-side comparison clearly indicates just how different the extent of motion is in the actual simulated microscopic ensemble as opposed to the one captured by the refinement. In particular, in the example of Trp23 (Fig. 6a–c), one can see how the refinement seriously underestimates the mobility of this side chain at the level of anisotropic thermal parameters. The likely cause could lie in the rigid-body, highly correlated, anharmonic motion of the entire side chain, which significantly contributes to the actual B-factors but is missed by both isotropic and anisotropic B-factor refinements whose central underlying premise is that of non-correlated motion with Gaussian character. Furthermore, in the case of His27 (Fig. 6g–i), the ring in the refined and starting model is flipped compared with the average structure, which shows how large the influence of the starting model is in the refinement. It is important to point out that the electron density is a probability distribution and it does not discern between the atoms that contribute to it. Therefore, it is possible to have cases where the electron density of a residue may be well defined, even though in reality it also populates conformations that significantly differ from the well-defined average. On the other hand, the contribution of these conformations to the overall fluctuations of the residue and its electron density will depend on how much they are actually represented in the ensemble. The advantage of an internally consistent simulation setup like the one used herein is precisely in that it allows for a complete access to this information, as discussed above.

## Discussion

Oversimplifications and difficulties associated with using B-factors to capture structural heterogeneity and dynamics of biomolecules in crystals have long been recognized^11^,^12^,^41^,^42^. This is especially true when it comes to identifying different contributions that go beyond the idealized definition of B-factors as a reflection of atomic mean-square displacements. In particular, rigid-body motions and lattice defects have been widely considered as important in this regard^28^,^43^. Here, we have performed one of the largest MD simulations of a protein crystal to date and have actively manipulated the level of its atomic fluctuations, while at the same time minimizing all other potential contributions to B-factors. Importantly, the approach we have taken is largely complementary to the goal of most MD studies of protein crystals^36^,^37^; instead of trying to reproduce experimental results or analyse the dynamics of proteins in the unit cell, we have used MD simulations to create a microscopically heterogeneous crystal (Figs 1 and 2) and then refined a model structure using its calculated structure factors, ensuring at the same time that the obtained results are not a consequence of the inadequacies and deficiencies sometimes associated with MD simulations (for example, force-field inaccuracies, low level of sampling, inappropriate treatment of boundary conditions or faulty choice of the water model)^44^,^45^. Indeed, given that our approach is internally self-consistent, our main findings remain fully valid even if the underlying ensembles in our simulations do not correspond to the real ensembles in physical villin headpiece crystals, which given our setup most likely do not.

Overall, we were able to show that the refined model, even at extremely low *R*_free_ values, cannot accurately reproduce the underlying microscopic diversity regardless of whether isotropic or anisotropic B-factors are used. Moreover, the agreement with the actual B-factors even in the low-to-medium B-factor range of 10–60 Å^2^ is qualitative at best with the refined B-factors frequently heavily underestimating the actual B-factors. Importantly, the observed disagreement increases both with a decrease in the resolution of the data used in refinement (Supplementary Fig. 4) and with an increase in the weights applied to the B-factor restraints regardless of the sigma values used for bonded atoms (Supplementary Fig. 5). Although we have kept the N-terminal part of the molecule position-restrained, there is still a possibility that the C-terminal part could be refined using the α_3_-helix as a TLS group. This might decrease the reported discrepancies, similarly to a study where each helix in a homo-tetramer of RM6 was divided into three TLS groups yielding a lower *R*_free_ factor in the refinement^24^. However, as the agreement between the refined and actual B-factor values is very high for backbone atoms throughout the molecule (Fig. 3a), it appears that most of heterogeneity indeed does stem from side-chain movements with very little backbone displacement, except for the last three C-terminal residues.

Most importantly, our results suggest that proteins in typical crystals may under some circumstances be more heterogeneous and dynamic than can be concluded from refined B-factors. What is more, estimates of both static and dynamic disorder in crystals can be equally affected: B-factors derived from ensemble-average structure factors (that is, those based on a single snapshot of the simulated crystal only) exhibit a similar level of discrepancy to those derived from both time- and ensemble-average structure factors (Supplementary Fig. 6). This finding agrees with the results of the pioneering work of Kuriyan *et al.*^11^ However, while in the original study the authors discussed the contribution of anisotropic motion as a major source of discrepancy between the actual and the refined B-factors, they never carried out anisotropic refinement. In fact, our results unequivocally demonstrate that even with anisotropic refinement the discrepancy between the actual and the refined B-factors remains large. To the contrary, we believe that our results more strongly support the possibility that the discrepancy is caused by a combination of conformational averaging and improper treatment of anharmonic, correlated motions as shown for several analytically tractable models of motion by Garcia *et al.*^12^ Recently, Janowski *et al.*^34^ have performed and analysed a set of large-scale unrestrained MD simulations of a crystal containing a helical decapeptide where they have observed excellent agreement between B-factors calculated from the simulations and the ones refined from the experimental data. However, they have detected discrepancies in the C-terminal region of the peptide that adopts alternative conformations and exhibits a higher degree of mobility than the rest of the molecule. In particular, they have noticed that for residues that adopt two alternate conformers, B-factors obtained from average structure factors may underestimate the true level of dynamics. Here, it is important to emphasize that, while in their case initial refinement only correctly modelled one of the two alternate conformers and erroneously attributed artificially low B-factors to it, we observe underestimation of the true level of dynamics even in cases where no dominant alternate conformers are present (for example, in the case of Trp23). This, importantly, is not in any way related to the fact that a part of the villin headpiece molecule in our simulations was position-restrained. In order to demonstrate this, we have created a 3 × 3 × 3 crystal from 216 randomly chosen, structurally heterogeneous villin headpiece conformers generated by fully unrestrained MD simulations, thereby obtaining a system that exhibits structural heterogeneity across all residues (Supplementary Fig. 7a). When we apply the same procedure for structure factor calculation, averaging and refinement to this system (details in Supplementary Table 3), we observe a similar level of discrepancy between actual and refined B-factors as in the case of partially restrained simulations (Supplementary Fig. 7b). This demonstrates that, in keeping with our original design, the use of position restraints simply prevents lattice defects and loss of symmetry, without in any way affecting our principal conclusions.

Overall, a potentially higher microscopic heterogeneity of protein crystals than what is captured by typical X-ray refinement could explain discrepancies between some NMR and X-ray structures such as the reported liquid-like core of ubiquitin, which was observed with NMR, but not in X-ray structures^23^. Moreover, higher-than-detected mobility of proteins in crystals could explain the paradoxical retention of enzymatic ability of some proteins in crystals^46^. However, it should be pointed out that when ensembles of structures were introduced into structure refinement, this phenomenon was captured with X-ray for certain proteins as well^47^. Finally, a potentially higher heterogeneity and dynamics in proteins also suggests a possible higher importance of conformational entropy in biomolecular processes than what is typically considered, agreeing with recent NMR and MD results^20^,^48^.

Furthermore, our results also relate to all the situations in which experimental B-factors have been compared against other estimates and measures of protein dynamics. For example, there have been several comparisons between experimental B-factors and fluctuations calculated via elastic network models^21^,^22^. Moreover, inverse proportionality between B-factors and the packing density of the neighbours of a given atom has been reported and explored^49^,^50^. Finally, there have been numerous MD studies in which atomic fluctuations from simulations have been directly compared with experimental B-factors with varying degrees of success^11^,^24^,^25^. It is possible that in all of these cases a part of the deviation from experiment actually stems from experimental models not accurately reporting on the level of true microscopic diversity as suggested by our results.

There are several important directions in which the present results should be extended. First, it would be interesting to see whether the effects of averaging would differ significantly in structure solution methods other than MR. Second, an extension in the direction of exploring the effects of disordered and ordered solvent molecules on the refinement process by using MD simulations would also be a logical next step. Third and most important, our results suggest that further improvements may be required when it comes to models used to describe the richness of atomic displacements in crystallographic refinement (as also suggested by other studies)^51^,^52^. However, the dangers in the application of complex models with a high number of parameters in refinement lie in possible over-fitting of the data^53^. There is obviously room for improvement in the refinement procedure, as shown in this study, and MD simulations could be the path to take. With the recent advances in the field, both at the hardware and software level^45^,^54^, rich structural ensembles of a given crystal could be readily created and used in the refinement similarly in spirit to Levin *et al.*^55^ and Burnley *et al.*^47^ We hope the results presented herein will provide motivation for further research in these directions.

## Methods

### MD simulations

X-ray structure of the villin headpiece domain (PDB code: 2F4K)^56^, a 35-residue protein, was used to create a crystal consisting of 27 unit cells (3 × 3 × 3) by first expanding the crystal from C222_1_ to P1 symmetry to obtain a unit cell containing eight molecules and then translating this unit cell to construct the large 3 × 3 × 3 crystal. The system was then simulated using the united-atom GROMOS 45A3 force field^57^ at pH 9, which is equivalent to zero net charge. As the structure also contained two norleucine residues, the parameters for these residues were obtained from the Vienna-PTM server^58^,^59^. Each simulation box (with dimensions: 59.03 × 119.70 × 225.27 Å) contained 216 protein molecules (eight molecules per unit cell and 373 atoms per protein) and 12,728 molecules of SPC^60^ water, adding up to 118,752 atoms in total. Energy of the simulated system was initially minimized in two cycles of steepest-descent energy minimization. The initial velocities for the atoms were taken from a Maxwell distribution at 100 K and the system was subsequently heated to 350 K in five steps of 50 K simulated for 20 ps at constant volume each. In parallel, atomic position restraints for the 14 C-terminal residues (161 atoms) of each protein molecule were uniformly relaxed (with the restraint spring constant going from 25,000−0 kJ mol^−1 ^nm^−2^ in steps of 5,000 kJ mol^−1 ^nm^−2^). The 21-N-terminal residues (212 atoms) were kept restrained throughout the equilibration and production runs using a restraint spring constant of 25,000 kJ mol^−1 ^nm^−2^. In the end, the system was equilibrated for additional 20 ps under the final conditions. The production simulations were run for 50 ns using the GROMACS 4.0.7 biomolecular simulation package^61^ with a 2-fs integration step and a coordinate output at every 10 ps. Constant volume conditions were employed to ensure that potential deformations of the lattice are avoided^62^. The solute and solvent were coupled separately to heat baths at 350 K using Berendsen’s thermostat with a relaxation time of 0.05 ps (ref. 63). Bond lengths were constrained using LINCS^64^, while van der Waals interactions were treated with a cutoff of 8 Å. Electrostatic interactions were computed using the particle mesh Ewald method^65^,^66^ with the direct sum cutoff of 8 Å and the Fourier spacing of ~1.2 Å using fifth-degree B-splines.

### Structural analyses

RMSD and RMSF values were calculated for each monomer in the simulated system by using *g_rms* and *g_rmsf* routines implemented in GROMACS but without performing least-squares fitting beforehand. In order to calculate RMSD and RMSF values and B-factors that are comparable to the ones obtained through the refinement procedure, every monomer was rotated and translated according to the crystal’s symmetry operators back to a reference position. This has yielded 1,080,000 monomers for which RMSF values and B-factors were calculated (equation (1)). These monomers were also used to calculate the average structure, which was used in MR, as well as the anisotropic B-factors (*g_rmsf* routine). Finally, side-chain χ angles were determined by using GROMACS routine *g_chi*.


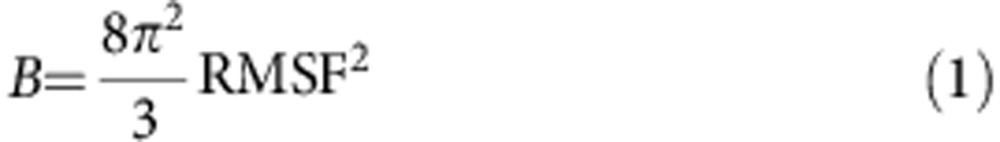


### Structure factor calculations and averaging

Output generated by the MD simulation contained 5,000 snapshots of the crystal, each consisting of 27 unit cells—that is, 216 protein molecules. Before structure factor calculations, water molecules were removed in order to simplify the system, expedite the calculations and restrict the observed effects exclusively to the structural disorder of the protein. Furthermore, every crystal structure was stripped of hydrogen atoms (which were added back later), and for each atom an occupancy of 1 and a B-factor of 15 Å^2^ were added in order to avoid singularities in Cromer–Mann structure factor tables that occur with low B-factor values. Structure factors for each crystal were calculated at 1.0 Å resolution in P1 space group by using a preparatory script from the diffraction simulator MLFSOM written by dr. James Holton ( http://bl831.als.lbl.gov/~jamesh/mlfsom/). The script used CCP4 (ref. 67) programs and the following protocol: (1) all hydrogen atoms were added using *hgen* that were modified to include the hydrogens for norleucines, (2) an electron density map was calculated using *sfall*^68^ with 5-Gaussian form for atomic scattering factors and 0.45 Å grid spacing for electron density sampling (132 × 266 × 500 grid points along unit cell axes), (3) the map was converted to structure factors by using *sfall* to create the final file. Structure factors calculated for 5,000 structures were then averaged (as vectors) and reduced to the C222_1_ space group by *sftools* resulting in 16,451 reflections in the resolution range of 37.5–1.0 Å. The added B-factors of 15 Å^2^ were removed by *cad* before refinement.

### X-ray refinement procedure

A total of 5% of average structure factors were randomly chosen for *R*_free_ calculations by *freerflag*^69^. The processed data were used in MR performed by *molrep*^70^ employing two different models: (1) the experimental villin headpiece structure (PDB code: 2F4K) stripped of water molecules and B rotamers (this was also the starting structure for MD simulations) and (2) the average structure calculated from all the conformations explored by the protein during the simulations. The latter is a nonphysical but geometrically more representative average depiction of the system. Multiple refinement runs were carried out by using *refmac5* (version 5.7.0029)^31^ and different combinations of the following settings: (1) B-factor refinement type (isotropic or anisotropic), (2) geometric restraining of the model (restrained or unrestrained) and (3) MR model (experimental 2F4K structure or the average one, as described above). The refinement ran for 25 cycles using default values for all other parameters, including automated weighting between geometry and X-ray target functions (where applicable). This was followed by 15 additional cycles with hydrogens added in riding positions for anisotropic, restrained refinement that used as the starting model in MR the experimental 2F4K structure.

## Author contributions

A.K., N.S.P. and B.Z. designed the study; A.K. performed the MD simulations, the calculations of structure factors and the refinements; A.K., N.S.P. and B.Z. analysed the data; A.K. wrote the manuscript; A.K., N.S.P. and B.Z. edited the manuscript.

## Additional information

**How to cite this article:** Kuzmanic, A. *et al.* X-ray refinement significantly underestimates the level of microscopic heterogeneity in biomolecular crystals. *Nat. Commun.* 5:3220 doi: 10.1038/ncomms4220 (2014).

## Supplementary Material

Supplementary Information Supplementary Figures 1-7, Supplementary Tables 1-3 and Supplementary References

## Figures and Tables

**Table 1:** Summary of the performed structural refinements.

***molrep*** **model**	**Starting structure**					**Average structure**			
**Type of refinement**	**Restrained**			**Unrestrained**		**Restrained**		**Unrestrained**	
**B-factor refinement**	**Iso**	**Aniso**	**Aniso**_**H**_	**Iso**	**Aniso**	**Iso**	**Aniso**	**Iso**	**Aniso**
*R* (%)	11.08	8.39	5.57	10.77	8.42	10.74	8	10.17	7.89
*R*_free_ (%)	11.21	8.97	5.85	11.36	9.38	10.75	8.22	10.55	8.77
RMS bond length (Å)	0.017	0.021	0.043	—	—	0.023	0.029	—	—
RMS bond angle (°)	2.101	2.537	3.870	—	—	2.895	3.400	—	—
RMS chiral volume (Å^3^)	0.150	0.155	0.260	—	—	0.301	0.244	—	—
RMS B-factors (Å^2^)	47.7	44.6	41.6	48.3	36.0	42.5	41.7	47.7	37.9
Δ_max_ (Å^2^)	336.6	348.3	318.4	357.9	332.1	345.4	325.3	346.7	292.0

Isotropic (Iso) and anisotropic (Aniso) refinements were performed using the average structure factors calculated from the simulated crystal structures (with 0.45 Å grid spacing for electron density sampling) for 25 cycles without hydrogens, while for anisotropic Aniso_H_ refinements 15 additional cycles were performed with hydrogens in riding positions. Root-mean-square (RMS) deviation is calculated for the following stereochemical quantities: bond lengths, bond angles and chiral volumes, as well as the deviation between the B-factor curves obtained from the simulations and the final model. The single largest difference (Δ_max_) between the B-factors obtained from the aforementioned curves is also reported.
