# Supplementary material for: X-ray refinement significantly underestimates the level of microscopic heterogeneity in biomolecular crystals
Source: Nat Commun. 2014 Feb 7;5:3220. doi: 10.1038/ncomms4220 (PMC3926004; doi:10.1038/ncomms4220)
Supplement: Supplementary Information — Supplementary Figures 1-7, Supplementary Tables 1-3 and Supplementary References [file ncomms4220-s1.pdf]

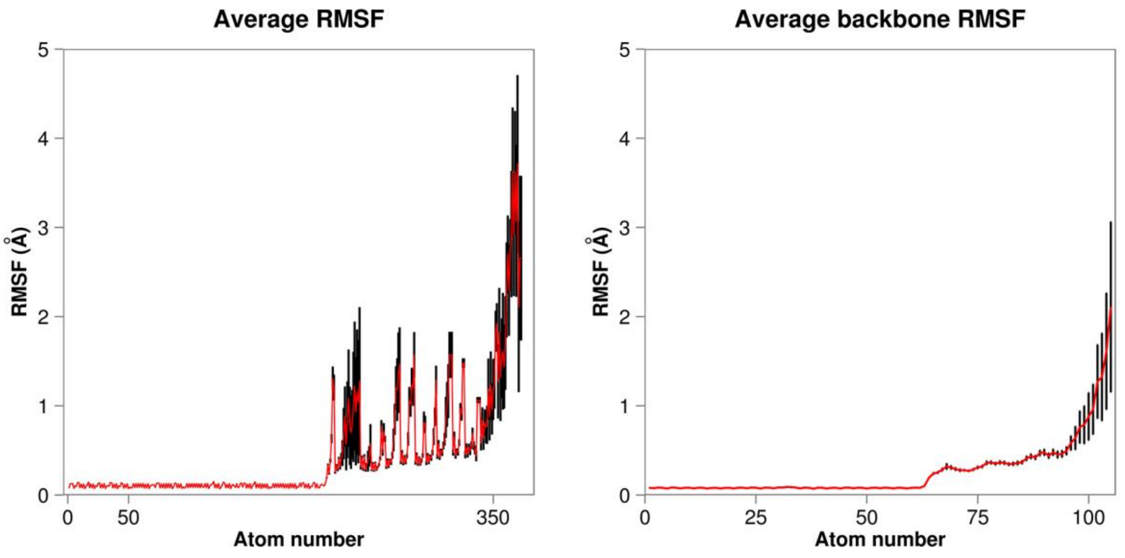

Supplementary Figure 1. Root-mean-square fluctuations (RMSF) profiles

Average all-atom RMSF (left) and backbone RMSF (right). The values shown have been averaged over 216 monomers in the crystal every 500 ps (red) and displayed together with their standard deviations (black). Only crystal-specific alignment has been applied prior to the calculations.

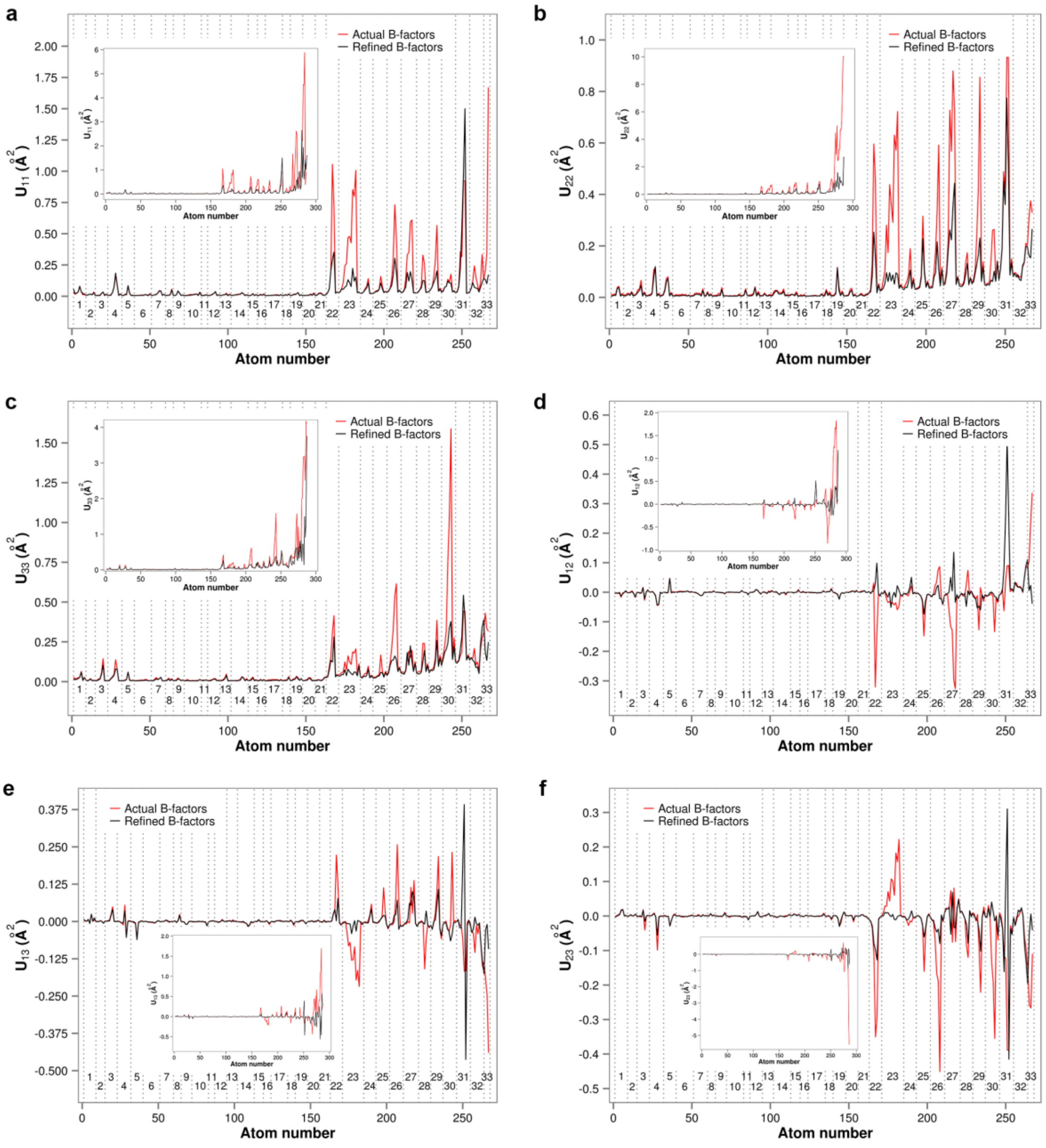

Supplementary Figure 2. Comparison of actual and refined anisotropic B-factors

Comparison of heavy-atom anisotropic B-factors calculated from MD simulation (red) with those obtained by crystallographic refinement (black) performed at the level of individual anisotropic displacement parameters: a) U_{11}, b) U_{22}, c) U_{33}, d) U_{12}, e) U_{13}, and f) U_{23}. The full area of each plot is shown in the inset, while the main graph excludes the last two residues. Individual residues have been labeled in principal plots and their boundaries indicated with grey dotted lines.

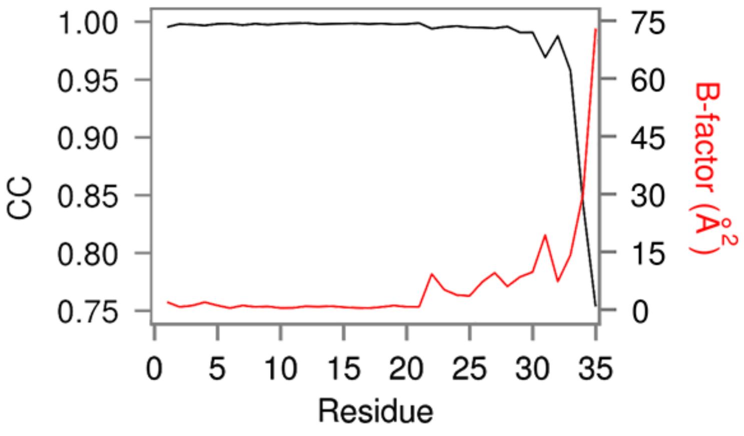

Supplementary Figure 3. Real-space correlation coefficients and refined B-factors

Real-space correlation coefficients (black) calculated with Phenix[1] for the data obtained in restrained anisotropic refinement solved by molecular replacement using the starting structure from the simulation as the initial model. Average B-factors for each residue are also shown (red).

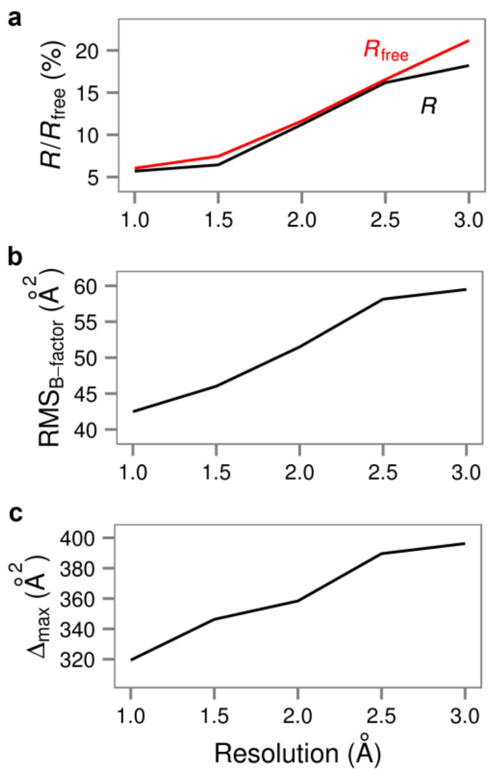

Supplementary Figure 4. Dependence of different parameters on the resolution of the data used in refinement

a) *R* and R_{free} factors, b) root-mean-square (RMS) deviation between the refined and actual B-factor profiles, and c) the largest difference between the refined and actual B-factors. Each refinement was performed for 25 cycles without hydrogens and for 15 cycles with hydrogens in riding positions, while the geometry was restrained and the B-factor refinement was anisotropic.

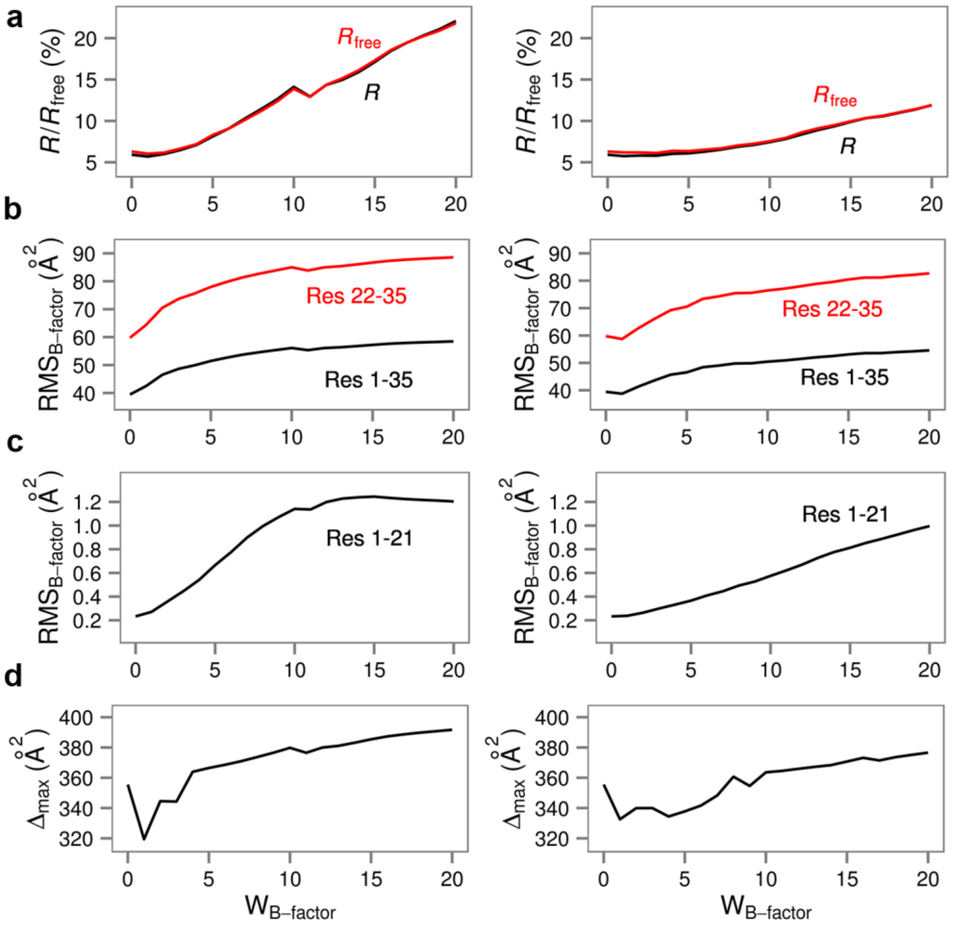

Supplementary Figure 5. Dependence of different parameters on the weights applied to B-factor restraints

a) R and R_{free} factors, b) and c) root-mean-square (RMS) deviation between the refined and actual B-factor profiles, and d) the largest difference between the refined and actual B-factors. Each refinement was performed for 25 cycles without hydrogens and for 15 cycles with hydrogens in riding positions. The geometry was restrained and the B-factor refinement was anisotropic. The σ values were set to 1.5 $Å^2$ and 2 $Å^2$ (main-chain bonds and angles, respectively), and to 3 $Å^2$ and 4.5 $Å^2$ (side-chain bonds and angles, respectively) for the graphs on the left, while for the graphs on the right, they were set to 5 $Å^2$ and 5.5 $Å^2$ (main-chain bonds and angles, respectively), and to 7 $Å^2$ and 8.5 $Å^2$ (side-chain bonds and angles, respectively) similarly to Tickle[2].

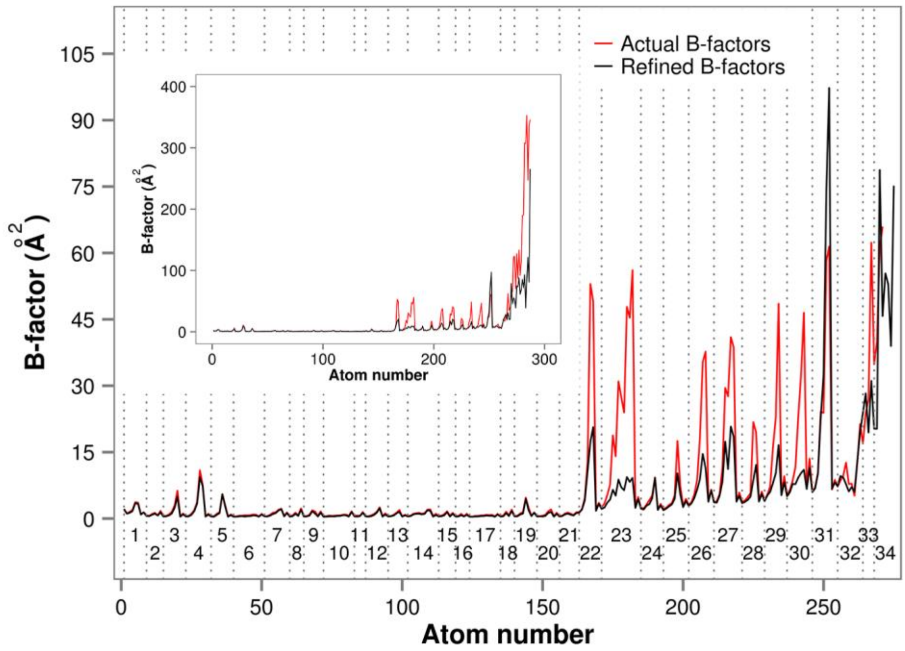

Supplementary Figure 6. Comparison of actual and refined B-factors

Comparison of heavy-atom B-factor profiles calculated from the last frame of the MD simulation (red) with those obtained by crystallographic refinement (black) for residues 1-34 and 1-35 (inset). Every residue in the main figure has been labeled and encased with grey dotted lines. The refinement procedure has been performed as described in the Methods section yielding R and R_{free} factors of 6.54% and 6.90%, respectively.

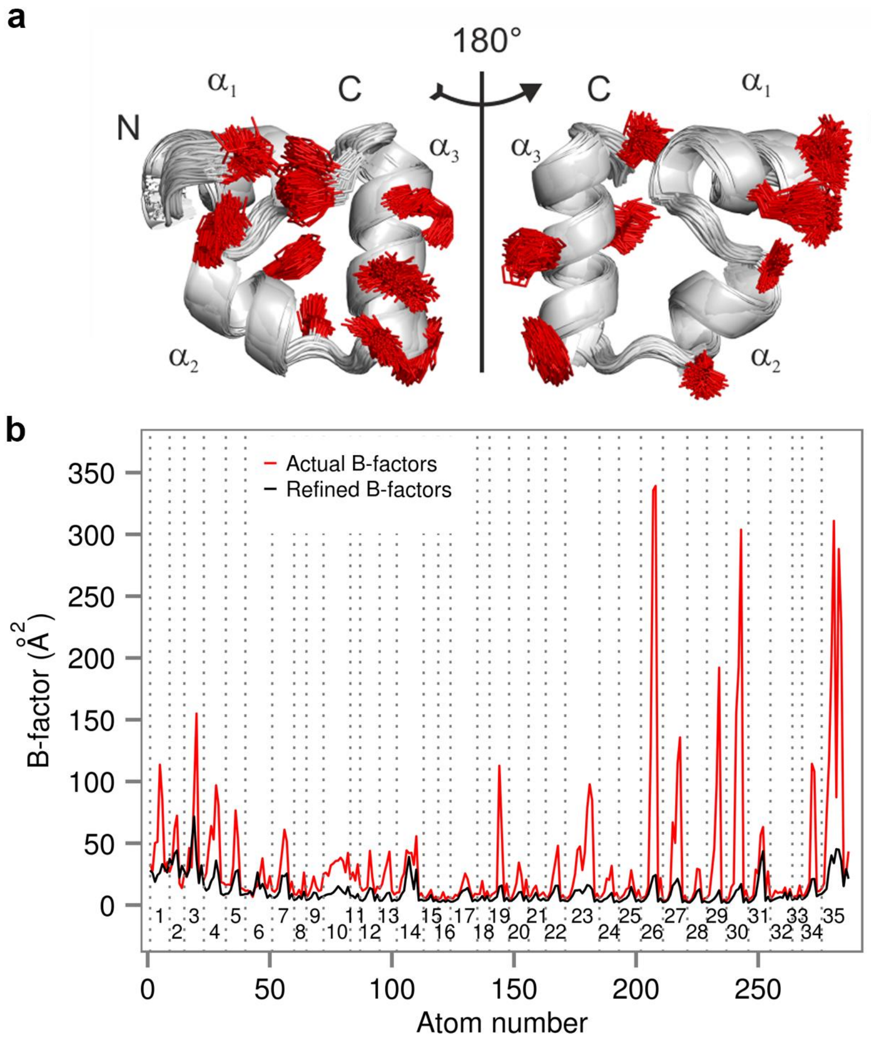

Supplementary Figure 7. Structural heterogeneity of villin headpiece domain crystal and the comparison of actual and refined B-factors obtained for the system

a) 216 villin headpiece structures randomly chosen from a fully unrestrained MD simulation of a single unit cell of the protein and used to create a 3x3x3 crystal by applying symmetry operators of $C222_1$ space group. The setup of the unrestrained simulations was the same as that for the partially restrained simulations described in the Methods section when it comes to all the relevant details. Selected side chains are shown in red (Leu1, Arg14, Phe17, Leu20, Leu22, Trp23, Gln26, Lys30, Phe35 on the left; Asp3, Glu4, Lys7, Thr13, Asn19, Trp23, His27, Nle29, Leu34 on the right). b) Comparison of heavy-atom B-factor profiles calculated for the 216 structures (red line) with those obtained by crystallographic refinement of the crystal (black line). Every residue has been labeled and encased with grey dotted lines.

Supplementary Table 1. Summary of the refinement performed using average structure factors calculated from the crystal structures obtained through MD simulations with 0.225 Å grid spacing for electron density sampling

molrep model	starting structure					average structure			
type of refinement	restrained			unrestrained		restrained		unrestrained	
B-factor refinement	iso	aniso	$aniso_H$	iso	aniso	iso	aniso	iso	aniso
R (%)	11.07	8.44	5.78	10.76	8.34	10.72	8.01	10.15	7.87
R_{free} (%)	11.21	8.95	6.06	11.36	9.08	10.75	8.29	10.54	8.74
RMS bond length (Å)	0.017	0.021	0.030	-	-	0.024	0.029	-	-
RMS bond angle (°)	2.101	2.508	3.336	-	-	2.924	3.309	-	-
RMS chiral volume (Å^3)	0.150	0.163	0.196	-	-	0.336	0.263	-	-
RMS B-factors (Å^2)	47.7	43.7	41.5	48.3	36.7	42.7	41.9	47.7	38.8
Δ_{max} (Å^2)	336.6	360.7	330.2	357.8	360.8	345.2	327.1	346.1	304.6

Refinements were performed for 25 cycles without hydrogens, while for $aniso_H$ refinement 15 additional cycles were performed with hydrogens in their riding positions. Root-mean-square (RMS) deviation is calculated for the following stereochemical quantities: bond lengths, bond angles and chiral volumes, as well as the deviation between the B-factor curves obtained from the simulations and the final model. The single largest difference (Δ_{max}) between the B-factors obtained from the aforementioned curves is also reported.

Supplementary Table 2. Summary of the refinement performed using structure factors calculated from the average density map obtained with 0.45 Å grid spacing

molrep model	starting structure
type of refinement	restrained
B-factor refinement	$aniso_H$
R (%)	5.61
R_{free} (%)	5.90
RMS bond length (Å)	0.034
RMS bond angle (°)	3.427
RMS chiral volume (Å^3)	0.211
RMS B-factors (Å^2)	41.56
Δ_{max} (Å^2)	306.9

Refinement was performed for 25 cycles without hydrogens and for 15 cycles with hydrogens in their riding positions. Root-mean-square (RMS) deviation is calculated for the following stereochemical quantities: bond lengths, bond angles and chiral volumes, as well as the deviation between the B-factor curves obtained from the simulations and the final model. The single largest difference (Δ_{max}) between the B-factors obtained from the aforementioned curves is also reported.

Supplementary Table 3. Summary of the refinement performed using structure factors calculated for the 3x3x3 crystal system created by applying symmetry operators to 216 diverse villin headpiece structures chosen randomly from the unrestrained simulations

molrep model	starting structure
type of refinement	restrained
B-factor refinement	$aniso_H$
R (%)	15.61
R_{free} (%)	18.24
RMS bond length (Å)	0.032
RMS bond angle (°)	2.926
RMS chiral volume ($Å^3$)	0.196
RMS B-factors ($Å^2$)	50.86
Δ_{max} ($Å^2$)	315.0

Refinement was performed for 25 cycles without hydrogens and for 15 cycles with hydrogens in their riding positions. Root-mean-square (RMS) deviation is calculated for the following stereochemical quantities: bond lengths, bond angles and chiral volumes, as well as the deviation between the B-factor curves obtained from the simulations and the final model. The single largest difference (Δ_{max}) between the B-factors obtained from the aforementioned curves is also reported.

Supplementary References

1. Adams, P. D., Afonine, P. V., Bunkoczi, G., Chen, V. B., Davis, I. W., et al. PHENIX: a comprehensive Python-based system for macromolecular structure solution. Acta Crystallogr. D 66(2), 213-221 (2010).

2. Tickle, I. Experimental determination of optimal root-mean-square deviations of macromolecular bond lengths and angles from their restrained ideal values. *Acta Crystallogr. D* **63**(12)**,** 1274-1281 (2007).
